# Complete Genome Sequences of Ralstonia solanacearum Strains Isolated from Infected Ginger Plants

**DOI:** 10.1128/mra.01298-22

**Published:** 2023-02-27

**Authors:** Mehbub Hasan, Miho Satoh, Akinori Kiba, Yasufumi Hikichi, Kouhei Ohnishi

**Affiliations:** a The United Graduate School of Agricultural Sciences, Ehime University, Matsuyama, Ehime, Japan; b Research Institute of Molecular Genetics, Kochi University, Nankoku, Kochi, Japan; c Faculty of Agriculture and Marine Science, Kochi University, Nankoku, Kochi, Japan; University of Strathclyde

## Abstract

We present the complete genome sequences of Ralstonia solanacearum strains isolated from ginger plants. Strains MAFF 211471, MAFF 211479, MAFF 211491, MAFF 301560, MAFF 241647, and MAFF 241648 contain 69, 64, 65, 69, 72, and 64 type III effector genes, respectively.

## ANNOUNCEMENT

Ralstonia solanacearum infects more than 200 plant species, including ginger plants, such as ginger, myoga, and kurukuma (1). The infection mechanism is mainly studied in *Solanaceae* plants (2). The infection mechanism seems to differ between dicots and monocots (3). In this study, we determine the complete genome sequences of several strains isolated from ginger.

The strains were obtained as freeze-dried cultures from the Research Center of Genetic Resources, NARO (Tsukuba, Japan). All six strains were isolated from infected ginger plants and are assigned as R. solanacearum by GenBank Project, NARO, using the nucleotide sequences of 16S rRNA genes and the *egl* gene. After adding 0.1 mL of sterilized water to lyophilize the dried samples, strains were streaked on B medium (1% Bacto peptone, 0.1% yeast extract, and 0.1% Casamino Acids) with 1.2% agar. Genomic DNA was extracted from cells grown overnight in B medium at 28°C using a Wizard HMW DNA extraction kit (Promega). The purified DNA was used for both short-read and long-read sequencing. For short-read sequencing, genomic DNA was fragmented and prepared with an average size of 330 bp using the QIAseq FX DNA library kit (Qiagen). After adaptors were ligated, the pooled libraries (at a concentration of 20 pM) were sequenced on an Illumina MiSeq (MiSeq Reagent kit v3, 150 cycles), generating 75-bp paired-end reads. Reads with Phred-encoded quality scores of more than 30 were selected for assembly. For long-read sequencing, genomic DNA was treated with the 1D Ligation Sequencing kit (SQK-LSK109; Oxford Nanopore Technologies [ONT]) with Native Barcoding Expansion (EXP-NBD104). After an adaptor was ligated, the pooled libraries were sequenced on MinION with Flow Cell (R9.4.1) under the control of MinKNOW (version 22.05.5; ONT). A fast model of base calling on MinKNOW was used, and the quality score was measured on the logarithmic Phred scale. Reads below the minimum quality score of 8 were removed as failed reads. The hybrid assembly with long and short reads was conducted using an assembly pipeline for bacterial genomes, Unicycler v0.4.7 (4). Unicycler produced complete circular replicons (5). Unicycler uses TBLASTN to search for the *dnaA* allele in each completed replicon, and the sequence was rotated. Gene annotation was conducted using DFAST prokaryotic genome annotation pipeline v.1.2.6 (6). Default parameters were used for all software unless otherwise specified. Characteristics of the sequenced genomes are listed in Table 1.

**TABLE 1:** Characterization of 6 R. solanacearum ginger strains

Strain	Accession no.	Genome size (bp)	GC content (%)	BioProject ID^*a*^	BioSample ID	Sequencer	DRA accession no.	Total no. of spots	Avg read length (bp)	*N*_50_ value (bp)	Coverage (×)
MAFF 211471 R. solanacearum		6,054,028	66.7	PSUB013500	SAMD00250057					3,914,648	83
Chromosome	AP024097	3,914,648				MiSeq	DRX406104	2,456,397			
Megaplasmid	AP024098	2,139,380				MinION	DRX406110	136,843	4,308	6,316	
MAFF 211479 R. solanacearum		5,905,596	66.8	PSUB013500	SAMD00250058					3,774,488	85
Chromosome	AP024099	3,774,488				MiSeq	DRX406105	1,646,478			
Megaplasmid	AP024100	2,131,108				MinION	DRX406111	155,208	5,554	9,943	
MAFF 211491 R. solanacearum		5,729,370	67.0	PSUB013500	SAMD00250059					3,635,021	87
Chromosome	AP024101	3,635,021				MiSeq	DRX406106	2,059,124			
Megaplasmid	AP024102	2,094,349				MinION	DRX406112	157,871	4,457	7,706	
MAFF 301560 R. solanacearum		5,762,965	66.9	PSUB013500	SAMD00250060					3,826,386	87
Chromosome	AP024103	3,826,386				MiSeq	DRX406107	1,819,899			
Megaplasmid	AP024104	1,936,579				MinION	DRX406113	180,022	4,534	6,659	
MAFF 241647 *R. solanacearum*		5,898,583	66.8	PSUB013500	SAMD00250061					3,820,899	85
Chromosome	AP024105	3,820,899				MiSeq	DRX406108	2,965,064			
Megaplasmid	AP024106	2,077,684				MinION	DRX406114	147,399	5,002	7,265	
MAFF 241648 *R. solanacearum*		5,887,878	66.7	PSUB013500	SAMD00250062					3,779,928	85
Chromosome	AP024107	3,779,928				MiSeq	DRX406109	2,691,832			
Megaplasmid	AP024108	2,107,950				MinION	DRX406115	131,716	3,958	5,715	

a ID, identifier.

The complete genome sequences of six R. solanacearum ginger strains indicated that all strains have two replicons, a chromosome and a megaplasmid, like other R. solanacearum strains (7). Bacterial type III effectors (T3Es) are one of the major pathogenic determinants (8) and important factors in determining host specificity (9). R. solanacearum ginger strains used in this study belong to phylotype I and form a distinct group from other phylotype I strains (10). T3Es were scanned at Ralsto T3E (9) (https://iant.toulouse.inra.fr/bacteria/annotation/site/prj/T3Ev3/) on 14 February 2020, and 69, 64, 65, 69, 72, and 64 T3Es, were detected in MAFF 211471, MAFF 211479, MAFF 211491, MAFF 301560, MAFF 241647, and MAFF 241648, respectively.

### Data availability.

This whole-genome shotgun (WGS) project has been deposited at DDBJ/EMBL/GenBank. The versions described in this paper are the first versions. The read archives have been deposited in DDBJ DRA. The genome nucleotide and DRA accession numbers are listed in Table 1.
